# Plasma exosomal IRAK1 can be a potential biomarker for predicting the treatment response to renin-angiotensin system inhibitors in patients with IgA nephropathy

**DOI:** 10.3389/fimmu.2022.978315

**Published:** 2022-08-26

**Authors:** Jianping Wu, Xiaona Wei, Jiajia Li, Yangang Gan, Rui Zhang, Qianqian Han, Peifen Liang, Yuchun Zeng, Qiongqiong Yang

**Affiliations:** Department of Nephrology, Sun Yat-sen Memorial Hospital, Sun Yat-sen University, Guangzhou, China

**Keywords:** IgA nephropathy, Renin-angiotensin system inhibitors, Exosome, Biomarker, Treatment response

## Abstract

**Background:**

Renin-angiotensin system inhibitors (RASi) are the first choice and basic therapy for the treatment of IgA nephropathy (IgAN) with proteinuria. However, approximately 40% of patients have no response to RASi treatment. The aim of this study was to screen potential biomarkers for predicting the treatment response of RASi in patients with IgAN.

**Methods:**

We included IgAN patients who were treatment-naive. They received supportive treatment, including a maximum tolerant dose of RASi for 3 months. According to the degree of decrease in proteinuria after 3 months of follow-up, these patients were divided into a sensitive group and a resistant group. The plasma of the two groups of patients was collected, and the exosomes were extracted for high-throughput sequencing. The screening of hub genes was performed using a weighted gene co-expression network (WGCNA) and filtering differentially expressed genes (DEGs). We randomly selected 20 patients in the sensitive group and 20 patients in the resistant group for hub gene validation by real-time quantitative polymerase chain reaction (qRT−PCR). A receiver operating characteristic (ROC) curve was used to evaluate hub genes that predicted the efficacy of the RASi response among the 40 validation patients.

**Results:**

After screening 370 IgAN patients according to the inclusion and exclusion criteria and the RASi treatment response evaluation, there were 38 patients in the sensitive group and 32 patients in the resistant group. IRAK1, ABCD1 and PLXNB3 were identified as hub genes by analyzing the high-throughput sequencing of the plasma exosomes of the two groups through WGCNA and DEGs screening. The sequencing data were consistent with the validation data showing that these three hub genes were upregulated in the resistant group compared with the sensitive group. The ROC curve indicated that IRAK1 was a good biomarker to predict the therapeutic response of RASi in patients with IgAN.

**Conclusions:**

Plasma exosomal IRAK1 can be a potential biomarker for predicting the treatment response of RASi in patients with IgAN.

## Introduction

IgA nephropathy (IgAN), the most common primary glomerular disease worldwide, is one of the major causes of chronic kidney disease (CKD) and end-stage renal disease (ESRD) (1). Proteinuria is a key factor affecting IgAN progression to ESRD (2). Renin-angiotensin system inhibitors (RASi) can effectively relieve proteinuria and delay renal insufficiency in patients with IgAN. RASi are the cornerstone of IgAN therapy (1). However, IgAN patients present different responses to RASi, and approximately half of IgAN patients is resistant to RASi treatment (3–7). There are currently no valuable predictive indicators or models to evaluate the treatment response of RASi in patients with IgAN.

Exosomes are double-membrane cystic vesicles secreted by a variety of cells. The bilayer membrane structure of exosomes can protect the RNA molecules within them from degradation by RNases, making them highly stable and specific (8). Previous studies have shown that exosome mRNA can be used as a biomarker to evaluate the treatment response of drugs with different diseases, such as cisplatin resistance in epithelial ovarian cancer (9), proteasome inhibitor resistance in multiple myeloma (10), gefitinib resistance to recipient cells (11), and patients’ responses to anti-programmed death-1 (PD-1) therapy (12).

In this study, we included a total of 370 patients in our hospital from March 2018 to September 2021. After screening according to the inclusion and exclusion criteria and the RASi treatment response evaluation, 38 patients were included in the sensitive group, and 32 patients were included in the resistant group. There were no differences in the clinical and pathological characteristics between the two groups. However, we found 3 hub genes through high-throughput sequencing of the plasma exosomes of the two groups. Ultimately, real-time quantitative polymerase chain reaction (qRT−PCR) verification and area under the curve (AUC) calculation showed that interleukin 1 receptor-associated kinase 1 (IRAK1) was the most relevant gene to the treatment response of RASi in patients with IgAN. Our findings suggest that plasma exosomal IRAK1 can be a potential biomarker to predict the treatment response of RASi in patients with IgAN. This study provides new evidence and strategies for individualized precision treatment of IgAN patients and provides new clues for optimizing the therapeutic effect of RASi therapy in other renal diseases.

## Materials and methods

### Patients and sample collection

**Figure 1:**
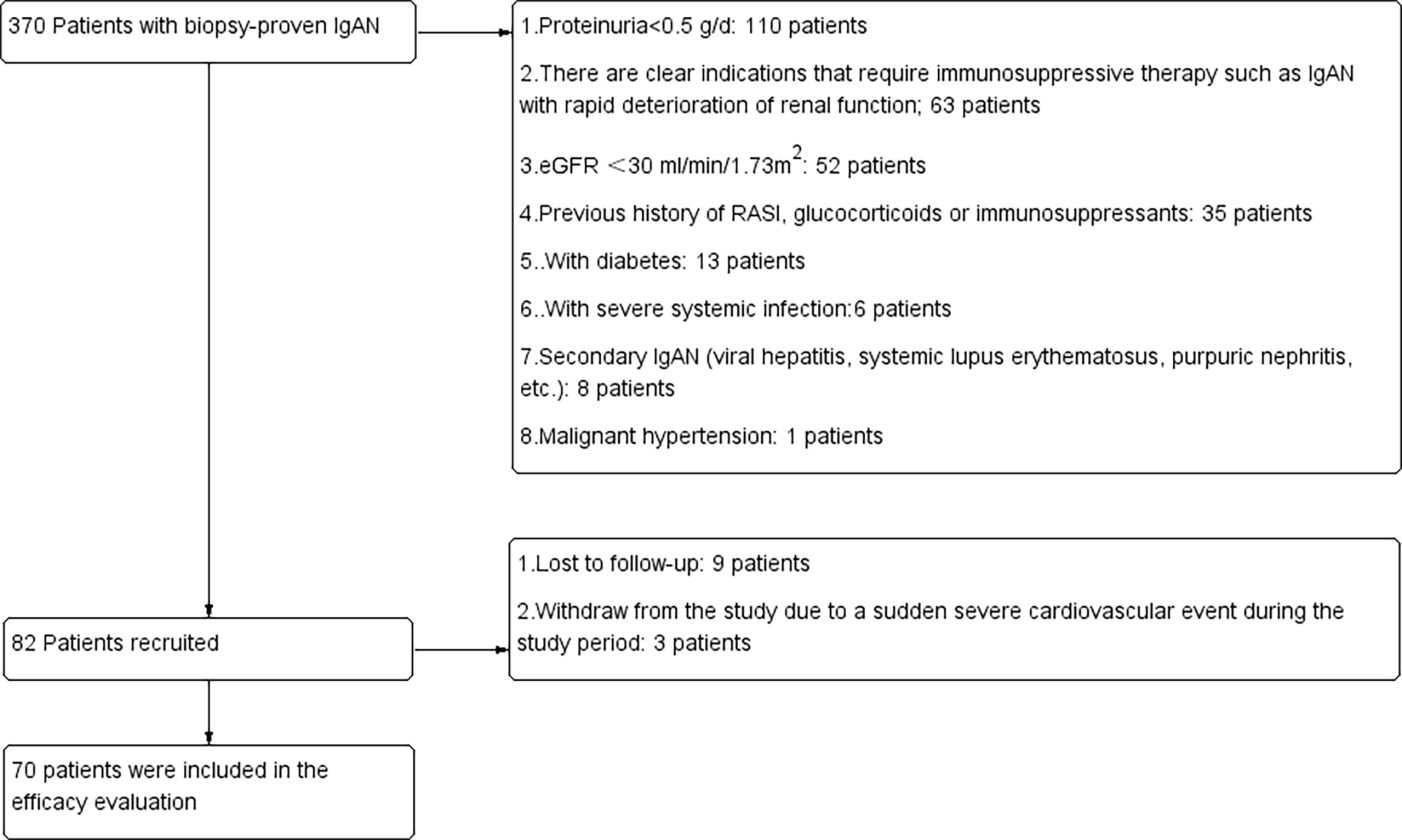
Details of patients screened, recruited and included in the final analysis. RASi, renin-angiotensin system inhibitors; eGFR, estimated glomerular filtration rate.

Patients who were treatment-naive and diagnosed with IgA nephropathy proven by pathological biopsy were recruited from the Department of Nephrology, Sun Yat-sen Memorial Hospital from March 2018 to September 2021. The inclusion criteria were as follows: 1. Patients who had been diagnosed with IgAN by renal biopsy and had not been treated with hormones or immunosuppressants; 2. The age of these patients was 18 years or older, and there were no gender restrictions; 3. 24-hour proteinuria > 0.5g/24h and estimated glomerular filtration rate(eGFR) ≥ 30 ml/min/1.73 m^2^. The exclusion criteria were as follows: 1. Secondary IgAN, such as systemic lupus erythematosus, allergic purpura, and hepatitis B-related nephritis; 2. There were clear indications for immunosuppressive therapy, such as IgAN with rapid deterioration of renal function; 3. eGFR <30 ml/min/1.73 m^2^; 4. Malignant hypertension; 5. Acute hepatitis and chronic active liver disease (aspartate aminotransferase, alanine transferase or bilirubin are 2.5 times higher than the upper limit of normal); 6. diabetes; 7. Systemic infection or history of severe infection within one month; 8. The existence of major organ diseases (such as severe cardiovascular disease, including congestive heart failure (CHF), chronic obstructive pulmonary disease, asthma that requires oral steroid therapy, central nervous system disease, etc.); 9. Those who had allergic reactions, contraindications or intolerance to RASi; 10. Those who were pregnant, breast-feeding, or used unreliable contraceptive methods; 11. Those who were unable or unwilling to provide written informed consent. All patients included in the study received the maximum tolerated dose of RASi for 3 months. After 3 months of treatment, the ratio of proteinuria decreased to ≥ 50% at baseline and was defined as the sensitive group, while < 50% was defined as the resistant group (7, 13, 14). The flowchart of the study is shown in **Figure 1**. This study was approved by the Ethical Review Committee of Sun Yat-sen Memorial Hospital (SYSEC-KY-KS-2018-080). All patients signed informed consent forms. All patients were fasted to draw 4 mL of venous blood and centrifuged to obtain plasma for future use.

### Extraction of exosomes and high-throughput RNA sequencing

Blood samples from 5 patients in the sensitive group and 5 patients in the resistant group were randomly selected for plasma exosome high-throughput RNA sequencing. The extraction of plasma exosomes was performed according to the procedure of the ExoQuick-TC kit’s instructions (System Biosciences, USA). Transmission electron microscopy (TEM) and flow cytometry were used to characterize the size and purity of exosomes. High-throughput RNA sequencing was performed by RiboBio (Guangzhou, China). We used principal component analysis (PCA) to check for outliers in the datasets, which was detailed in **Supplement Figure 1**. The high-throughput sequencing was performed based on the platform of illumina nova. Clean reads were obtained after filtering and global trimming by Fastp (v0.23.0) (15). The reads were normalized by using the “estimateSizeFactors” function in the DESeq2 package(v1.25.9) (16). Hisat2 (version 2.1.0) was used to align clean reads to the human genome (UCSC/HG19). After integrating the location information of the genome corresponding to all reads, the reads were mapped on the exonic, intronic, and intergenic of chromosome (17).

### Hub gene screening by DEGs screening and WGCAN

DEGs screening was performed by the “limma” package from R (18). Genes satisfying the conditions of adjusted P value <0.10 and | log2(fold change) |> 0.58 were defined as DEGs between the sensitive group and resistance group (19, 20). All DEGs were drawn as heatmaps with the R package “pheatmap”. The WGCNA network was constructed by using the R package “WGCNA” (21). The module and sample feature correlation heatmap and module and sample feature hierarchical clustering map were constructed to determine the key module that had the highest correlation coefficient with treatment response. Genes that met the criteria that the value of gene significance (GS) was greater than 0.2 and module membership (MM) was greater than 0.8 were identified as hub genes (22).

### qRT−PCR

qRT−PCR was used to verify the hub genes. Total RNA of exosomes was extracted by a miRNeasy Mini kit (Qiagen, Germany), cDNA synthesis was performed by using a reverse transcription kit (Takara, Japan), and qRT−PCR was performed on the Bio-Rad CFX Connect (Bio-Rad, California, USA). Primer sequences are detailed in **Supplementary Material Table 1**.

### ROC

The ROC curve of the hub genes was drawn using the pROC package (23) of R. We evaluated the predicting treatment response of the hub genes by calculating the AUC. When the AUC was greater than 0.8, the hub genes were defined as having a relatively ideal predictive value (24).

### Statistics

SPSS 23.0 statistical software was used for statistical analysis. Measurement data conforming to the normal distribution was expressed as the means ± standard deviation. Comparisons between the two groups were performed by t test. Data that did not conform to a normal distribution was represented by quartiles. The classification mapping was completed by Prism GraphPad 6.0 software. The percentage (%) was used to count data, and the chi-square test was used for comparison and analysis between groups. *P < 0.05* indicates that the difference is statistically significant.

## Results

### Clinical and pathological characteristics of patients in the sensitive group and resistance group

From March 2018 to March 2021, 370 patients were screened in the Department of Nephrology, Sun Yat-sen Memorial Hospital, Sun Yat-sen University; 288 patients were excluded because they did not meet the inclusion and exclusion criteria. Nine patients were lost to follow-up, and patients withdrew from the study due to a sudden severe cardiovascular event during the study period. Finally, 70 patients were included in the final analysis (**Figure 1**). According to the degree of decrease in proteinuria after 3 months of follow-up, there were 36 patients in the resistance group and 32 patients in the resistance group. The general demographic information, laboratory tests and pathological characteristics of these patients are detailed in **Table 1**. There were no significant differences between the sensitive group and the resistant group in sex, age, degree of proteinuria or pathological manifestations.

**Table 1:** Demographic, clinical and pathological characteristics of the sensitive and resistant groups.

Characteristic	Sensitive group (n=38)	Resistance group (n=32)	*P* value
**Age (yr)**	**36.66±11.6**	**36.22±10.39**	**0.87**
**Male sex (%)**	**18 (47.37)**	**20 (62.50)**	**0.47**
**BMI**	**22.78±2.75**	**23.78±3.23**	**0.17**
**SystolicBP (mm Hg)**	**102.13±22.13**	**103.28±19.96**	**0.82**
**Diastolic BP (mm Hg)**	**85.11±11.09**	**84.62±9.66**	**0.85**
**24-h Urinary protein (g)**	**1.54±0.85**	**1.43±0.75**	**0.59**
**Hemoglobin (g/L)**	**135±15.45**	**127±17.41**	**0.25**
**Albumin (g/L)**	**36.04±5.73**	**37.6±5.90**	**0.27**
**Cholesterol (mmol/L)**	**5.17±1.24**	**5.38±1.17**	**0.47**
**Triglycerides (mmol/L)**	**1.69±1.44**	**1.58±1.04**	**0.73**
**High density lipoprotein (mmol/L)**	**1.22±0.31**	**1.26±0.32**	**0.56**
**Low density lipoprotein (mmol/L)**	**3.25±0.90**	**3.45±0.82**	**0.33**
**Calcium (mmol/L)**	**2.28±0.11**	**2.28±0.12**	**0.99**
**Phosphorus (mmol/L)**	**1.17±0.17**	**1.18±0.13**	**0.85**
**Alanine aminotransferase (IU/L)**	**17.6±10.72**	**20.68±12.18**	**0.27**
**Aspartate aminotransferase(IU/L)**	**18.66±5.8**	**19.88±5.86**	**0.39**
**Creatinine (umol/L)**	**114.37±56.26**	**100.75±55.91**	**0.32**
**Blood urea nitrogen (mmol/L)**	**6.29±3.20**	**5.68±2.55**	**0.39**
**Uric acid (mmol/L)**	**429.05±121.29**	**415.47±107.21**	**0.62**
**eGFR (ml/min per 1.73 m2)**	**73.58±27.22**	**81.96±25.61**	**0.19**
**M1 (%)**	**37 (97.37)**	**31 (96.87)**	**0.71**
**El (%)**	**9 (23.68)**	**7 (21.88)**	**0.86**
**S1 (%)**	**26 (68.42)**	**20 (62.50)**	**0.60**
**T1/2 (%)**	**11 (28.95)**	**11 (34.38)**	**0.56**
**C1 (%)**	**23 (60.53)**	**15(46.88)**	**0.25**

BMI, body mass index; BP, blood pressure; eGFR, estimated glomerular filtration rate.

### Identification of exosomes

**Figure 2:**
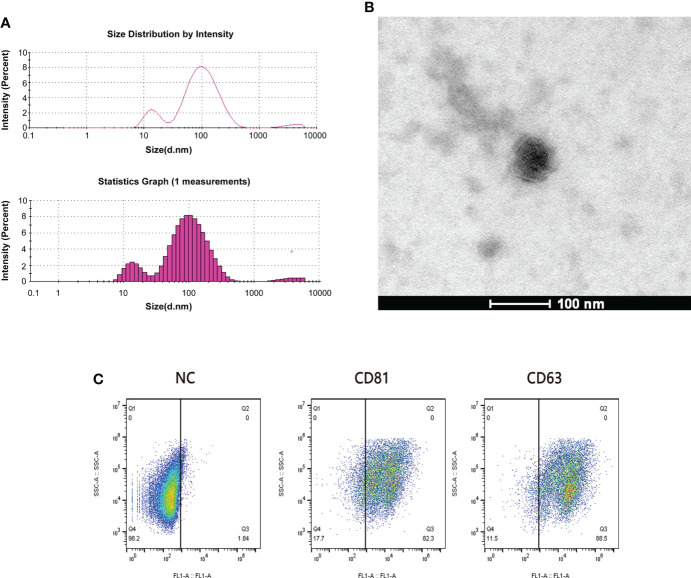
Quality examination of plasma exosomes. **(A)** The average particle size of exosomes was measured by dynamic light scattering (DLS). **(B)** The form of plasma exosomes was observed by transmission electron microscopy (TEM). Scale bar = 100 nm. **(C)** The purity of plasma exosomes was evaluated through the flow cytometry calculating the positive rates of CD81 and CD63.

We randomly selected 5 patients from the sensitive group and the resistant group for plasma exosome transcriptome sequencing. We performed the identification of exosomes by using TEM and flowcytometry as refer to our previous study (25). Dynamic light scattering (DLS) analysis showed that the average particle size of the extracted exosomes was 62.68 nm (**Figure 2A**). This is consistent with the results observed by TEM (**Figure 2B**). Exosomes retain relatively complete antigens or receptors on the surface, and the common specific marker proteins of exosomes are CD63 and CD81 (26). We judged the purity of exosomes by flow cytometry, and the results showed that the positive rates of CD63 and CD81 were 88.5% and 82.3%, respectively (**Figure 2C**).

### Hub gene screening by DEGs screening and WGCAN

**Figure 3:**
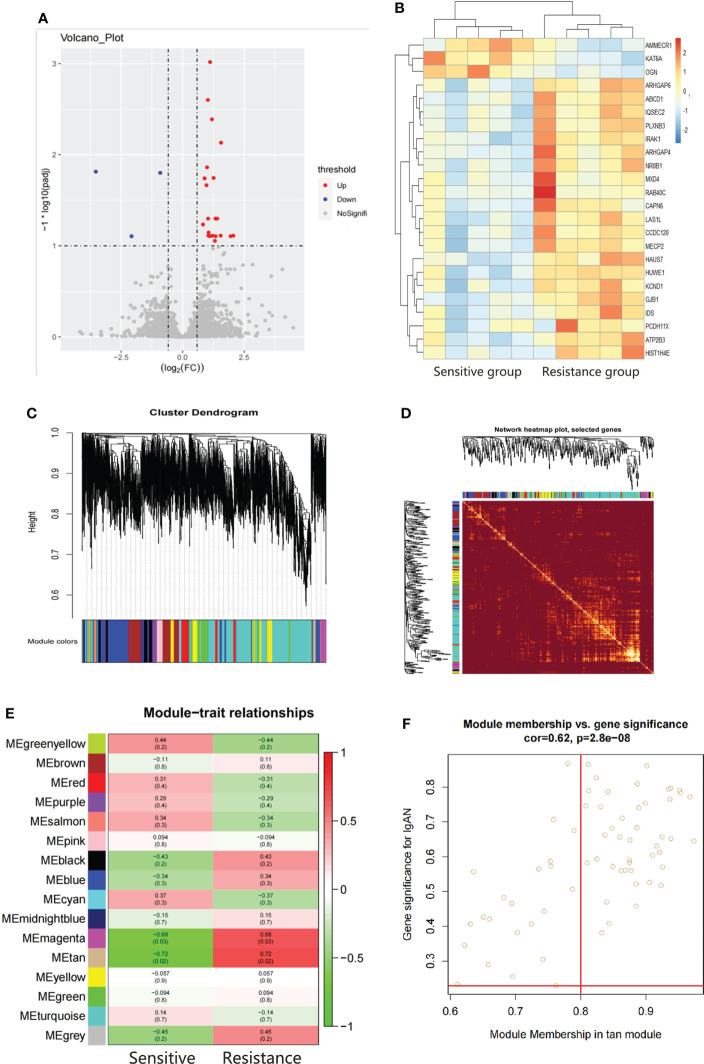
Hub gene screening by DEGs screening and WGCAN. **(A, B)** Volcano plot and heatmap of DEGs between the sensitive group (n=5) and the resistant group (n=5). **(C)** Gene dendrogram. **(D)** Heatmaps visualizing 400 randomly selected genes in the network to depict the topological overlap matrix. **(E)** Heatmap of module–trait relationships. **(F)** The distribution of GS and MM was presented as a scatter diagram in the tan module.

**Figure 4:**
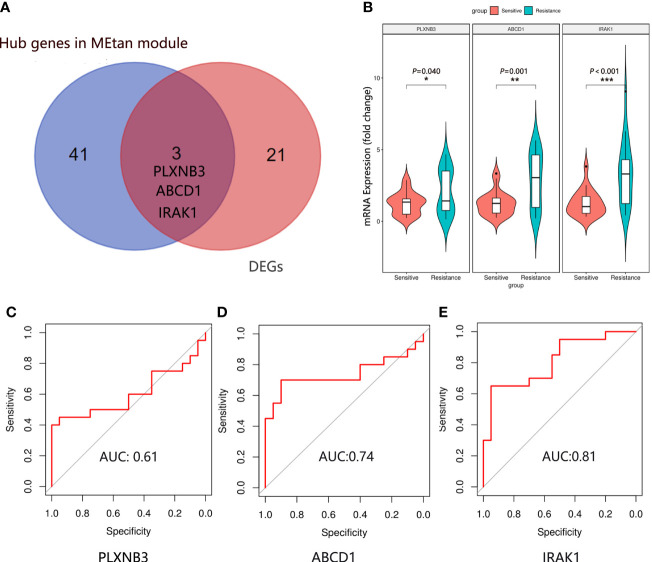
Hub gene identification and validation. **(A)** The final hub genes were determined by Venn diagram. **(B)** Expression of IRAK1, ABCD1 and PLXNB3 mRNA in the plasma exosomes in the sensitive group (n=20) and resistance group (n=20). Mean ± SEM, *P < 0.05, **P < 0.01, ***P < 0.001. The ROC curve of IRAK1 **(C)**, ABCD1 **(D)** and PLXNB3 **(E)** in predicting the RASi response in IgAN.

There were 24 DEGs identified, including 21 upregulated genes and 3 downregulated genes after screening the DEGs based on this condition, the resistance group was compared with the sensitive group (**Figures 3A, B**). There were sixteen gene co-expression modules in the WGCNA (**Figure 3C**). Overall, 400 genes were randomly selected for heatmap display **(** **Figure 3D**). The sixteen gene co-expression modules and RASi response heatmap showed that the tan module had the most significant correlation with RASi response (**Figure 3E**). Genes that met the criteria that the value of gene significance (GS) was greater than 0.2 and module membership (MM) was greater than 0.8 were determined to be hub genes (**Figure 3F**). Hub genes found in both the WGCNA and DEGs, namely, IRAK1, ABCD1 and PLXNB3, were identified as the final hub genes (**Figure 4A**).

### Validation of hub genes

We randomly selected 20 patients in the sensitive and resistant groups for hub gene validation by real-time quantitative polymerase chain reaction (qRT−PCR). We found that the 3 genes were upregulated in the resistant group compared with the sensitive group (PLXNB3, *P = 0.040*; ABCD1, *P = 0.001*; IRAK1, *P* < 0.001), which was consistent with the sequencing results (**Figure 4B**).

### Evaluation of hub genes for the predictive effectiveness of RASi treatment response in IgAN

To determine which gene is the most predictive of the treatment response of RASi with IgAN, a receiver operating characteristic (ROC) curve was used to evaluate the genes predicting the therapeutic response of RASi in the treatment of IgAN with proteinuria based on the qRT−PCR results of the 40 patients. We evaluated the predictive effectiveness of each gene by calculating the area under the curve. The AUC of PLXNB3 was 0.61 (**Figure 4C**), that of ABCD1 was 0.74 (**Figure 4D**) and that of IRAK1 was 0.81 (**Figure 4E**).

## Discussion

Proteinuria is one of the main clinical manifestations of IgA nephropathy, and remission of proteinuria can improve its prognosis (27). RASi drugs can dilate glomerular efferent arterioles, inhibit mesangial cell proliferation and reduce matrix secretion to protect the kidney from injury (28). Clinical guidelines recommend RASi for the treatment of IgA nephropathy if the patient with proteinuria >0.5 g/d, indicating that RASi is the cornerstone of the initial treatment with IgAN (1). There are large individual differences in the treatment response of RASi with IgAN patients. Previous studies have shown that only approximately 40%-60% of patients can effectively relieve proteinuria after RASi treatment (3–7). In our study, we also found that 46% of IgAN patients showed a response to RASi. Recently, a prospective study including 96 IgAN patients found that the resistance group was characterized by more severe clinical and histological manifestations than the sensitive group, which was different from our study in which we found no clinical or pathological differences between the sensitive group and the resistant group (7). The possible reasons were that the race was different, and our patient was characterized by milder renal pathology than the patients in Bagchi et al. (7). On the other hand, our inclusion and exclusion criteria were stricter, and all patients with pure IgAN were included, which could have excluded the interference of other confounding factors and better evaluated the treatment response of RASi.

The RNA contained in exosomes has higher specificity and integrity, can more accurately reflect changes in renal function and structural damage and is more suitable as a marker for evaluating the treatment response of drugs with different diseases (29). Since there were no differences in the clinical and pathological characteristics of the patients between the sensitive group and resistance group, we randomly selected the plasma exosomes of 5 patients from the two groups for high-throughput sequencing. WGCAN and DEGs screening are powerful data analysis tools that can identify potential biomarkers of great intervention and therapeutic value in different diseases (30). We identified the cluster genes most related to the RASi treatment response with IgAN by WGCAN and filtered the DEGs between the two groups. There were 44 genes in the WGCNA and 24 hub genes among the DEGs. The genes found in both the WGCNA and DEG analyses were defined as the final hub genes; IRAK1, ABCD1 and PLXNB3 were identified as the hub genes related to the RASi treatment response in IgAN. We randomly selected 20 patients in the sensitive and resistant groups for hub gene validation by qRT−PCR. The results indicated that these three hub genes were upregulated in the resistant group compared with the sensitive group. Consistent with the sequencing data, ROC analysis showed that IRAK1 but not ABCD1 or PLXNB3 had good predictive performance.

IRAK1, a member of the IRAK family, plays an important role in inflammatory autoimmune diseases. The underlying mechanism is that IRAK1 binds to TLRs to initiate downstream inflammatory pathways, such as mitogen-activated protein kinase (MAPK) and NF-κB (31). In sepsis-related studies, IRAK1 knockout mice showed less inflammation and higher survival rates than normal control mice (32). Multiple clinical studies have found that polymorphisms of the IRAK1 gene are associated with prognosis in sepsis patients (33, 34). In kidney-related studies, it was found that the expression of IRAK1 in the peripheral blood of patients with lupus nephritis was increased, and the use of IRAK1 inhibitors could alleviate kidney damage in lupus mice (35). In a mouse model of diabetic nephropathy, downregulation of IRAK1 could inhibit kidney damage in mice by inhibiting the apoptosis of podocytes and thereby reducing proteinuria (36). Stem cell therapy ameliorated renal injury in mice with ischemia−reperfusion and was found to be associated with decreased IRAK1 expression (37). Recent studies have shown that IRAK1 is associated with drug treatment response. Li et al. (38) found that IRAK1 drives tumor resistance to radiotherapy by activating the downstream PIN1 signaling axis. IRAK1 expression is increased in breast cancer paclitaxel-resistant cell lines, an IRAK1 inhibitor reverses paclitaxel resistance in triple-negative breast cancer, and IRAK1 is also a potential therapeutic target for paclitaxel resistance in nasopharyngeal carcinoma (39). There is currently no research related to IRAK1 in resistance to RASi in IgAN patients, and its potential mechanism needs further study.

This research has several highlights. First, we analyzed the sensitivity to RASi treatment in IgAN patients and compared the clinical and pathological characteristics of the patients between the sensitive group and the resistant group. Second, we analyzed the high-throughput sequencing data of exosomes through bioinformatics analysis and identified exosomal IRAK1 as a potential biomarker for predicting the therapeutic response of RASi with IgAN. However, our clinical sample size is still relatively small, and the prediction efficiency of IRAK1 for the RASi response within IgAN patients has not been validated in other centers.

## Conclusion

In summary, 46% of IgAN patients showed resistance to RASi in our cohort, and there were no differences in the clinical or pathological characteristics of the patients between the sensitive group and the resistant group. By analyzing and validating exosome transcriptome sequencing data from the two groups, we identified exosomal IRAK1 as a potential biomarker for predicting the therapeutic response of renin-angiotensin system inhibitor in IgAN patients with proteinuria.

## Data availability statement

The datasets presented in this study can be found in online repositories. The name of the repository and accession number can be found below: NCBI Sequence Read Archive; SRP385967.

## Ethics statement

The studies involving human participants were reviewed and approved by the Ethical Review Committee of Sun Yat-sen Memorial Hospital (SYSEC-KY-KS-2018-080). The patients/participants provided their written informed consent to participate in this study.

## Author contributions

QY contributed to the conception of the study. JW and XW performed the experiment. JL, RZ, QH, PL and YZ performed the data analyses and wrote the manuscript. All authors contributed to the article and approved the submitted version.

## Funding

This project was funded by the National Key R&D Programme of China (2021YFC2009404); the Science and Technology Projects in Guangzhou (201904010142) and the Natural Science Foundation of Guangdong Province (2021A1515010801).

## Conflict of interest

The authors declare that the research was conducted in the absence of any commercial or financial relationships that could be construed as a potential conflict of interest.

## Publisher’s note

All claims expressed in this article are solely those of the authors and do not necessarily represent those of their affiliated organizations, or those of the publisher, the editors and the reviewers. Any product that may be evaluated in this article, or claim that may be made by its manufacturer, is not guaranteed or endorsed by the publisher.
