# Corrigendum: Plasma exosomal IRAK1 can be a potential biomarker for predicting the treatment response to renin-angiotensin system inhibitors in patients with IgA nephropathy

**DOI:** 10.3389/fimmu.2022.1074864

**Published:** 2022-11-07

**Authors:** Jianping Wu, Xiaona Wei, Jiajia Li, Yangang Gan, Rui Zhang, Qianqian Han, Peifen Liang, Yuchun Zeng, Qiongqiong Yang

**Affiliations:** Department of Nephrology, Sun Yat-sen Memorial Hospital, Sun Yat-sen University, Guangzhou, China

**Keywords:** IgA nephropathy, Renin-angiotensin system inhibitors, Exosome, Biomarker, Treatment response

In the published article, the funding information was missing. The correct Funding statement appears below.

## Funding

This project was funded by the National Key R&D Programme of China (2021YFC2009404); the Science and Technology Projects in Guangzhou (201904010142) and the Natural Science Foundation of Guangdong Province (2021A1515010801).

The authors apologize for this error and state that this does not change the scientific conclusions of the article in any way. The original article has been updated.

## Publisher’s note

All claims expressed in this article are solely those of the authors and do not necessarily represent those of their affiliated organizations, or those of the publisher, the editors and the reviewers. Any product that may be evaluated in this article, or claim that may be made by its manufacturer, is not guaranteed or endorsed by the publisher.

