# Supplementary figures and images for: Plasma exosomal IRAK1 can be a potential biomarker for predicting the treatment response to renin-angiotensin system inhibitors in patients with IgA nephropathy
Source: Front Immunol. 2022 Aug 26;13:978315. doi: 10.3389/fimmu.2022.978315 (PMC9459338; doi:10.3389/fimmu.2022.978315)

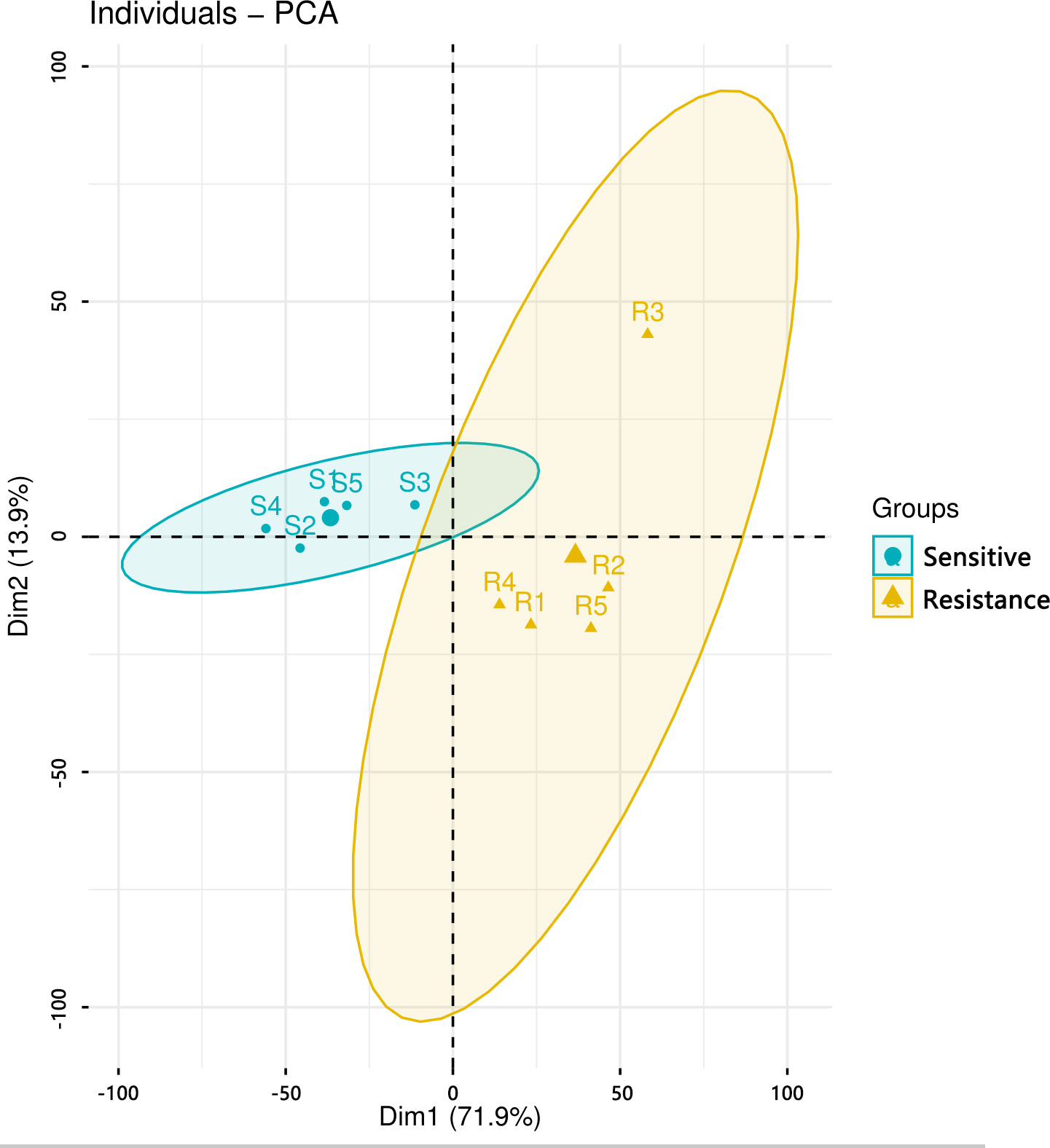

Supplement: Supplementary file 1 [file Image_1.jpeg]
