# Supplementary material for: Plasma exosomal IRAK1 can be a potential biomarker for predicting the treatment response to renin-angiotensin system inhibitors in patients with IgA nephropathy
Source: Front Immunol. 2022 Aug 26;13:978315. doi: 10.3389/fimmu.2022.978315 (PMC9459338; doi:10.3389/fimmu.2022.978315)
Supplement: Supplementary file 2 [file Table_1.docx]

| genes | Sequences |
| --- | --- |
| Homo GAPDH | Forward：5‘-TGTGGGCATCAATGGATTTGG-3’ |
|  | Reverse：5‘-ACACCATGTATTCCGGGTCAAT-3’ |
| Homo IRAK1 | Forward：5‘-ATGTCTCTGCAAAAGACCCCT-3’ |
|  | Reverse：5‘-GGTAGCAGTTTGGGCTTGTGT-3’ |
| Homo ABCD1 | Forward：5‘-GCTGGCATGAACCGGGTATT-3’ |
|  | Reverse：5‘-GCCACATACACCGACAGGAA-3’ |
| Homo PLXNB3 | Forward：5‘-CGCTTCTCCGCACCTAATACC-3’ |
|  | Reverse 5‘-CAGGGCTGTCGATTACAGGG-3’ |
